# Proviruses with identical sequences comprise a large fraction of the replication-competent HIV reservoir

**DOI:** 10.1371/journal.ppat.1006283

**Published:** 2017-03-22

**Authors:** John K. Bui, Michele D. Sobolewski, Brandon F. Keele, Jonathan Spindler, Andrew Musick, Ann Wiegand, Brian T. Luke, Wei Shao, Stephen H. Hughes, John M. Coffin, Mary F. Kearney, John W. Mellors

**Affiliations:** 1 Division of Infectious Diseases, Department of Medicine, University of Pittsburgh School of Medicine, Pittsburgh, Pennsylvania, United States of America; 2 Howard Hughes Medical Research Fellows Program, Howard Hughes Medical Institute, Bethesda, Maryland, United States of America; 3 AIDS and Cancer Virus Program, Frederick National Laboratory for Cancer Research operated by Leidos Biomedical Research, Inc., Frederick, Maryland, United States of America; 4 HIV Dynamics and Replication Program, National Cancer Institute, Frederick, Maryland, United States of America; 5 Advanced Biomedical Computing Center, Frederick National Laboratory for Cancer Research operated by Leidos Biomedical Research, Inc., Frederick, Maryland, United States of America; 6 Department of Molecular Biology and Microbiology, Tufts University, Boston, Massachusetts, United States of America; University of Illinois at Chicago College of Medicine, UNITED STATES

## Abstract

The major obstacle to curing HIV infection is the persistence of cells with intact proviruses that can produce replication-competent virus. This HIV reservoir is believed to exist primarily in CD4^+^ T-cells and is stable despite years of suppressive antiretroviral therapy. A potential mechanism for HIV persistence is clonal expansion of infected cells, but how often such clones carry replication-competent proviruses has been controversial. Here, we used single-genome sequencing to probe for identical HIV sequence matches among viruses recovered in different viral outgrowth cultures and between the sequences of outgrowth viruses and proviral or intracellular HIV RNA sequences in uncultured blood mononuclear cells from eight donors on suppressive ART with diverse proviral populations. All eight donors had viral outgrowth virus that was fully susceptible to their current ART drug regimen. Six of eight donors studied had identical near full-length HIV RNA sequences recovered from different viral outgrowth cultures, and one of the two remaining donors had identical partial viral sequence matches between outgrowth virus and intracellular HIV RNA. These findings provide evidence that clonal expansion of HIV-infected cells is an important mechanism of reservoir persistence that should be targeted to cure HIV infection.

## Author summary

Antiretroviral therapy (ART) can reduce plasma HIV viral loads to undetectable levels. However, HIV-infected cells can persist despite years of antiretroviral suppression and contribute to rebound viremia following cessation of ART. It is unclear whether HIV-infected cells are maintained by ongoing viral replication, prolonged survival of infected cells, or clonal expansion of infected cells. No studies to date have definitively determined whether clonal expansion is a common mechanism of persistence for replication-competent HIV. To investigate, we used single-genome sequencing to detect identical HIV sequence matches between replication-competent viruses, proviruses, and/or cell-associated RNA from eight donors who were on long-term suppressive ART without evidence of ongoing viral replication. Six of eight donors had identical near full-length sequences between unique replication-competent viruses. One of the two remaining donors had identical partial viral sequences between a replication-competent virus and cell-associated HIV RNA. These findings suggest that clonal expansion is an important mechanism of persistence of replication-competent HIV and should be targeted by HIV cure efforts.

## Introduction

Antiretroviral therapy (ART) rapidly suppresses HIV replication [1], but it is not curative because cells carrying intact (replication-competent) proviruses persist and lead to rebound viremia when ART is stopped [2–9]. This HIV reservoir is very stable with an estimated half-life of 44 months [2–6]. Clonal expansion of HIV-infected cells may be an important mechanism that contributes to the stability of the HIV reservoir [10–12], but this concept is controversial and has not been convincingly demonstrated to be a common persistence mechanism of the replication-competent reservoir [12–15].

We sought additional evidence that cells carrying replication-competent proviruses can clonally expand *in vivo*. We probed for identical replication-competent viral sequences from HIV RNA in the viral outgrowth assay (VOA) and from proviral DNA and/or cell-associated RNA in blood mononuclear cells by using single-genome sequencing (SGS) targeting the p6-PR-RT amplicon [16]. Identical partial sequence matches from different VOA wells were confirmed by near full-length genome sequencing. Recovery of identical sequences from different VOA wells from donors with diverse proviral populations strongly suggests the clonal expansion of infected cells carrying replication-competent proviruses.

## Results

Experiments were performed using samples from eight chronically HIV-1 infected adults on long-term suppressive ART (plasma HIV RNA <50 HIV copies/mL for >2 years) who did not have outgrowth viruses with resistance mutations to the corresponding donor’s ART regimen. The characteristics of the eight donors are summarized in Table 1 and S1 Table. Donors had a median duration of viremia suppression on ART (<50 HIV RNA copies/mL) of 7 years (range 4–15). Median residual plasma viremia, measured by the single-copy assay [16], was 1.6 RNA copies/mL (range <0.4 to 13.7). The median nadir CD4 count was 259 cells/mm^3^ (range 80–410) and the median current CD4 count was 878 cells/mm^3^ (range 357–1578). All donors began ART during the chronic stage of HIV infection; median average pairwise distance (APD) of proviral sequences was 1.0% (range 0.5–2.2, Table 2) [17]. Median total HIV DNA level, measured by qPCR [18], was 915 copies per million CD4^+^ T-cells (range 164–2401). Median cell-associated HIV RNA, measured by qRT-PCR [18], was 279 (range 38–713). Median infectious units per million resting CD4^+^ T-cells (IUPM), measured by VOA [19], was 0.44 (range <0.1 to 2.5).

**Table 1:** Characteristics of Study Donors. Study donors were a convenience sample of eight chronically HIV-1 infected adults on suppressive ART with plasma HIV RNA less than 50 copies/mL for longer than two years.

Donor ID	Age	Sex	Race	Estimated years infected	Years on suppressive ART	Plasma HIV RNA (copies / mL)	Nadir CD4 (cells / mm^3^)	Current CD4 (cells / mm^3^)	HIV DNA (copies / 10^6^ tCD4)	Cell-associated HIV RNA (copies / 10^6^ tCD4)	Infectious Units per Million Cells (IUPM)
1	53	M	CA	4	4	2.2	237	948	1724	500	tCD4: 0.65
											rCD4: 2.5
2	49	M	CA	11	11	3.8	306	1100	660	96	tCD4: 1.6
											rCD4: 0.65
3	46	F	CA	22	6	< 0.4	272	520	164	138	tCD4: 1.6
											rCD4: < 0.1
4	32	M	CA	11	7	13.7	259	1578	958	713	tCD4: 0.1
											rCD4: < 0.21
5	57	M	AA	27	5	1.3	127	807	872	252	tCD4: 0.32
											rCD4: 0.22
6	54	M	AA	15	> 6	1.9	N.A.	357	328	38	tCD4: N.D.
											rCD4: 0.76
7	52	F	AA	22	10	<0.6	80	699	2401	321	tCD4: N.D.
											rCD4: 0.12
8	56	F	AA	26	15	<0.6	410	1288	1066	305	tCD4: N.D.
											rCD4: 1.38
**Median**	52.5	5M, 3F	4 CA, 4 AA	18.5	7	1.6	259	878	915	279	rCD4: 0.44

tCD4, total CD4^+^ T-cells; rCD4, resting CD4^+^ T-cells; M, Male; F, Female; CA, Caucasian-American; AA, African-American; N.A., not available; N.D., not done.

**Table 2:** Frequencies of identical replication-competent proviral sequences.

Donor ID	APD of proviral sequences* (%)	Fraction of replication-competent proviruses that have sequence matches^†^ in p6-PR-RT	Identical replication-competent proviruses confirmed by near full-length sequences	Description of HIV sequence matches observed^‡^
1	0.5	6/8 (75.0%)	Yes	• Identical sequences in 6 VOA wells by p6-PR-RT SGS and overlapping half-genome sequencing. Linked near full-length sequences are intact.
2	1.0	6/10 (60.0%)	Yes	• Identical sequences in 2 VOA wells by p6-PR-RT SGS and overlapping half-genome sequencing with 1 matching identical proviral sequence by p6-PR-RT SGS. Linked near full-length sequences are intact. • Identical sequences (distinct from above) in 3 VOA wells by p6-PR-RT SGS and overlapping half-genome sequencing. Linked near full-length sequences are intact.
3	1.2	4/8 (50.0%)	Yes	• Identical sequences in 2 VOA wells by p6-PR-RT SGS and overlapping half-genome sequencing. Linked near full-length sequences are intact. • Identical sequences (distinct from above) between 1 VOA well and 2 proviruses by p6-PR-RT SGS. Linked near full-length sequence is intact. • Identical sequences (distinct from above) between 1 VOA well and 4 proviruses by p6-PR-RT SGS.
4	1.0	5/21 (23.8%)	Yes	• Identical sequences in 3 VOA wells by p6-PR-RT SGS and overlapping half-genome sequencing. Linked near full-length sequences are intact. • Identical sequences (distinct from above) between 1 VOA well and 1 provirus by p6-PR-RT SGS.
5	2.0	0/9 (< 11.1%)	Not Done	• No sequence matches observed.
6	0.7	3/3 (100.0%)	Yes	• Identical sequences in 2 VOA wells by p6-PR-RT SGS and overlapping half-genome sequencing. Linked near full-length sequences are intact. • Identical sequences (distinct from above) between 1 VOA well and 2 proviruses by p6-PR-RT SGS. Linked near full-length sequence is intact.
7	0.6	1/1 (100.0%)	Not Done	• Identical sequences between 1 VOA well and 1 cell-associated HIV RNA molecule by p6-PR-RT SGS. Linked near full-length sequence is intact.
8	2.2	8/15 (53.3%)	Yes	• Identical sequences between 1 VOA well and 1 VOA well from a separate time point by p6-PR-RT SGS and overlapping half-genome sequencing. Linked near full-length sequences are intact. • Identical sequences (distinct from above) between 1 VOA well and 1 VOA well from a separate time point by p6-PR-RT SGS. Linked near full-length sequence is intact. • Identical sequences (distinct from above) between 1 VOA well and 1 VOA well from a separate time point by p6-PR-RT SGS. Linked near full-length sequence is intact. • Identical sequences (distinct from above) between 1 VOA well and 1 VOA well from a separate time point by p6-PR-RT SGS. • Identical sequences (distinct from above) between 2 VOA wells from the same time point by p6-PR-RT SGS. Linked near full-length sequence is intact.
**Median**	1.0	56.7%		

APD, average pairwise distance; SGS, single-genome sequencing; VOA, viral outgrowth assay.

*Average pairwise distances determined using HIV DNA p6-PR-RT sequences, excluding sequences with hypermutant signatures.

^†^Sequence matches between individual p24-positive VOA wells or between p24-positive VOA wells and HIV DNA or cell-associated HIV RNA.

^‡^‘Linked’ near full-length sequences are defined as near full-length sequences that match p6-PR-RT SGS sequences. ‘Intact’ sequences are defined as not containing large deletions, frame-shift mutations, or disabling stop codons.

**Fig 1:**
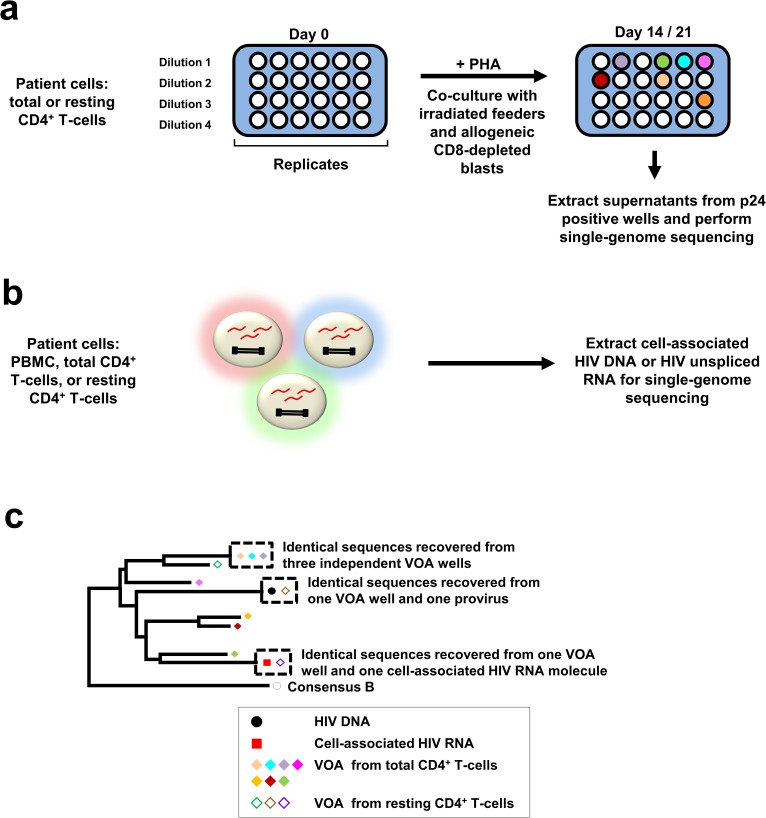
Schematic of experimental approach. *(a)* To perform quantitative viral outgrowth assays (VOA), donor CD4^+^ T cells (resting or total) were serially diluted, stimulated with PHA, and co-cultured with irradiated feeder cells and CD8-depleted allogeneic blasts from HIV-negative donors for 14 to 21 days. Single-genome sequencing (p6-PR-RT or near full-length) was performed on supernatants from p24-positive wells, and the sequences from the different wells were analyzed for identical matches. *(b)* Single-genome sequencing was performed on uncultured blood mononuclear cells to obtain p6-PR-RT sequences from HIV DNA and/or unspliced HIV RNA to search for identical sequence matches to RNA sequences from p24-positive VOA wells. *(c)* Schematic showing hypothetical examples of identical sequence matches between single-genome sequences from p24-positive wells, HIV DNA, and cell-associated HIV RNA analyzed by neighbor-joining distance analysis.

We identified sequences from replication-competent HIV by performing the VOA on total or resting CD4^+^ T-cells derived from whole blood (Fig 1). Resting CD4^+^ T-cells were defined as CD4^+^ T-cells that are CD69^-^CD25^-^HLA-DR^-^. VOA wells were considered to have replicating virus if they had a positive p24 EIA by day 14 or 21 of the VOA. Single-genome sequencing (SGS) of the genomic region encoding p6 of gag, protease, and the first ~900 nucleotides of reverse transcriptase (p6-PR-RT; nt 1893–3408, HXB2 positions [20]) was performed to screen for identical sequences between p24-positive VOA supernatants and HIV DNA and/or cell-associated HIV RNA extracted from multiple, independent aliquots of peripheral blood mononuclear cells (PBMC), total CD4^+^ T-cells, or resting CD4^+^ T-cells purified from uncultured blood (Fig 1) [21, 22]. The presence of identical p6-PR-RT sequences in multiple VOA wells suggests clonal expansion of cells containing replication-competent proviruses, wherein each VOA well was seeded with at least one cell from the clone. Because different cell aliquots were used to perform the VOA, HIV DNA SGS, and cell-associated HIV RNA SGS, the presence of sequences from VOA wells that match sequences from HIV DNA or cell-associated HIV RNA also suggests clonal expansion of cells carrying replication-competent proviruses. When identical p6-PR-RT sequences were found across multiple VOA wells, near full-length single-genome sequences of virion-associated HIV RNA were obtained from the VOA wells to confirm viral sequence identity (S1 Fig) [23]. This approach offers improved sensitivity for the detection of rare cells carrying identical, replication-competent proviruses (~2% of all proviruses) [24–27] as compared to prior methods that employed integration site analysis with linkage to proviral sequences [12, 13].

To obtain near full-length viral sequences from p24-positive VOA wells, SGS was performed on single viral RNA templates to amplify and sequence 5’ and 3’ overlapping half-genomes (S1 Fig). Near full-length sequences were constructed by linking 5’ and 3’ half-genome sequences using their overlapping region. Each VOA well had 1–3 dominant viral sequences that could be discerned from one another by the overlapping genomic region. Viral replication in the VOA can introduce mutations (1 per 10,000–100,000 bases per replication cycle) [28, 29] and RT-PCR is known to generate errors primarily from the RT step (1–2 per 10,000 bases) [21]. To identify such errors, consensus near full-length sequences were derived from the half-genome sequences obtained from single templates.

We assayed a maximum 13 million cells for each VOA. The median frequency of HIV-infected cells among the donors was 915 HIV DNA-containing cells per million CD4^+^ T-cells (Table 1), thus each VOA assayed ~12,000 infected cells. Approximately 2% of infected cells (or ~240) were expected to contain intact proviruses [27]. Based upon having near full-length sequences that are ~9 kb long with a median average pairwise distance (APD) of 1.0% (Table 2), the probability of detecting two identical proviruses in the assayed cells, assuming no clonal expansion had occurred, is <10^−23^ by Poisson probability distribution (see Methods). Therefore, identical near full-length sequence matches from two different VOA wells strongly suggest clonal expansion of HIV-infected cells.

**Fig 2:**
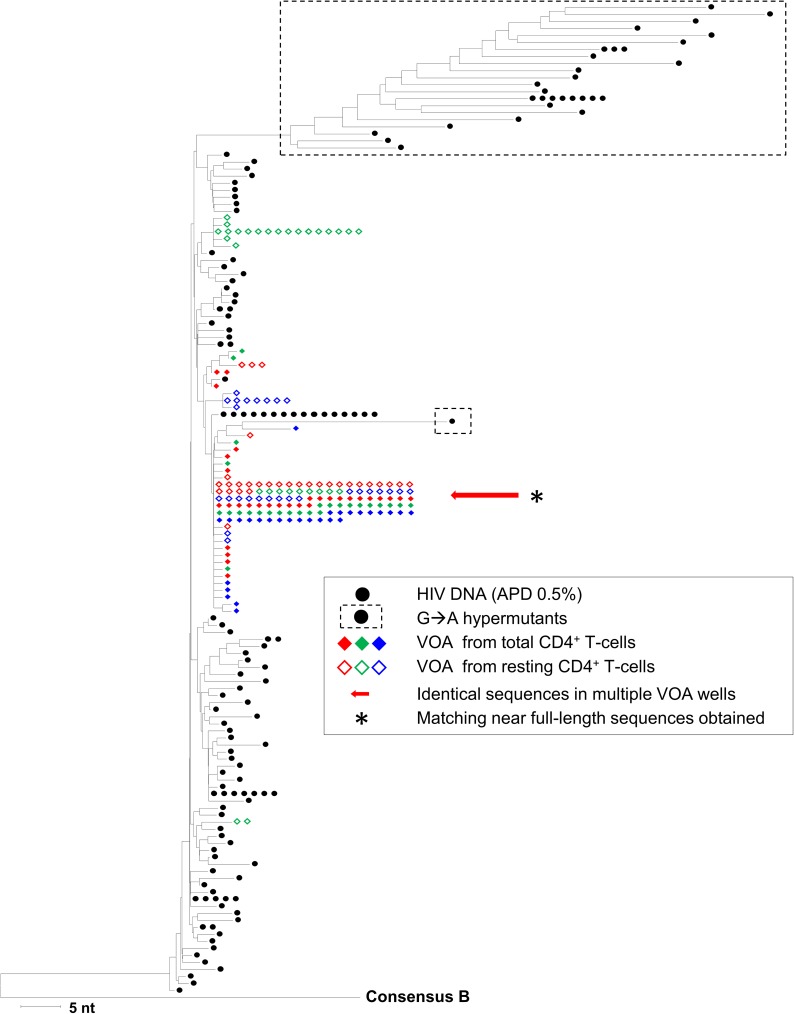
Neighbor-joining distance tree of sequences in p24-positive viral outgrowth assay wells and in HIV DNA sequences from blood mononuclear cells (Donor 1). The tree was constructed using the neighbor-joining p-distance method and rooted to a subtype B consensus sequence. p6-PR-RT single-genome sequences were obtained from HIV DNA in total CD4^+^ T-cells. Hypermutant HIV DNA sequences are shown in dashed boxes. p6-PR-RT single-genome sequences were also obtained from independent, p24-positive viral outgrowth assay (VOA) wells performed using either total CD4^+^ T-cells or resting CD4^+^ T-cells. Different colored diamond symbols represent sequences from different p24-positive VOA wells. Identical p6-PR-RT sequences were recovered from six p24-positive VOA wells (red arrow), with confirmed matches of viral RNA by overlapping half-genome sequencing (*). These matching near full-length sequences appeared intact without large deletions, frame-shift mutations, or disabling stop codons. Note that the large groups of identical sequences from VOA wells are accompanied by multiple sequence variants that differ from the predominant sequence by 1–2 nucleotides, a result we attribute to mutations that arose during *ex vivo* virus replication and/or to errors introduced during *in vitro* cDNA synthesis.

Fig 2 shows a representative neighbor-joining distance tree of p6-PR-RT sequences from Donor 1. Identical sequences were recovered from six independent, p24-positive VOA wells (Fig 2, red arrow). Note that all groups of identical sequences from the p24-positive VOA wells in this and subsequent figures are accompanied by minor sequence variants differing by 1–2 nucleotides from the main group, which we attribute to mutations that accumulated during *ex vivo* viral replication in the VOA and/or from errors introduced during *in vitro* cDNA synthesis [21]. The identity of the intact proviruses in all six VOA wells was confirmed by near full-length viral genome sequencing (Fig 2, asterisk), which is highly unlikely to occur by chance sampling of identical proviruses from a population without clonal expansion (P <10^−100^ by Poisson probability distribution, see Methods).

A similar result was obtained with samples from Donor 2 (S2 Fig). Identical p6-PR-RT sequences were recovered from two independent, p24-positive VOA wells and a single provirus (S2 Fig, blue arrow). Identical near full-length HIV sequences were also obtained from both VOA wells. A second group of identical p6-PR-RT sequences was recovered from four other p24-positive VOA wells (S2 Fig, red arrow). Identical near full-length HIV sequences were obtained from three of the four wells. The probability of sampling the observed identical near full-length proviruses from this population assuming no clonal expansion is <10^−100^ by the Poisson probability distribution (see Methods).

Overall, identical p6-PR-RT sequence matches between multiple VOA wells were found in samples from six of eight donors (Table 2): Donor 1 (Fig 2), Donor 2 (S2 Fig), Donor 3 (S3 Fig), Donor 4 (S4 Fig), Donor 6 (S6 Fig), and Donor 8 (S8 Fig). For these six donors, identical p6-PR-RT sequences across VOA wells were confirmed with near full-length sequences. None of the near full-length sequences contained large deletions, frame-shift mutations, or disabling stop codons. No evidence for recombination between sequences in p24-positive VOA wells was found by analyses using SimPlot [30]. Coreceptor tropism analysis of *env* by Geno2Pheno [31] implied that the outgrowth virus had variable tropism (S1 Table).

Of the two other donors studied, one had identical p6-PR-RT sequences from one VOA well and one cell-associated HIV RNA molecule (Donor 7, S7 Fig). The near full-length HIV sequence obtained from this VOA well that linked to the matching p6-PR-RT sequences did not contain large deletions, frame-shift mutations, or disabling stop codons. In the remaining donor (Donor 5, S5 Fig), we did not find any identical p6-PR-RT sequences across VOA wells or between VOA wells and HIV DNA.

A summary of all observed sequence matches among VOA wells or between VOA wells and HIV DNA or cell-associated HIV RNA in all donors is provided in Table 2. More than half (median 57%) of the replication-competent viruses isolated had identical sequence matches in other VOA wells, HIV DNA, or cell-associated HIV RNA. Significant correlations were not found between the prevalence of clones and the parameters in Table 1, although power to detect associations was limited by the small sample size. Five of eight donors did not have detectable proviral sequences in blood mononuclear cells that matched VOA sequences (range <0.9% to 6.1%, S2 Table). In all donors, multiple, identical p6-PR-RT sequences were observed in HIV DNA and/or cell-associated HIV RNA that did not match VOA sequences.

## Discussion

The primary barrier to an HIV cure is the stable latent reservoir of HIV-infected cells that can produce replication-competent virus and contribute to rebound viremia following ART cessation [2–9]. Multiple mechanisms of HIV persistence have been proposed, including ongoing viral replication, prolonged survival of HIV-infected cells, and clonal expansion. Several pieces of evidence refute the possibility that viral replication occurs in patients on appropriate ART therapy. Using sensitive evolutionary tests, studies have shown no evidence of viral evolution in donors during long-term suppressive ART [8, 9, 32]. Furthermore, treatment intensification studies have not shown effects on residual viremia [33–35].

Clonal expansion of infected cells was first proposed as a mechanism for the oligoclonal nature of residual viremia [32, 36]. Subsequent studies using integration site analysis have found that infected cells can undergo clonal proliferation, but these studies did not determine the replication-competence of the proviruses within clones [10, 11]. Conflicting reports have since been published on whether infected cells carrying replication-competent proviruses undergo clonal expansion *in vivo*. Cohn *et al*. did not detect intact proviral sequences from 75 clones that they claimed to have identified in eight donors [12]. Bruner *at al*. found that all 38 near full-length clones recovered from 19 donors contained gross defects precluding replication [15]. By contrast, Simonetti *et al*. reported on an HIV-infected individual with a highly expanded clone carrying an intact provirus responsible for persistent, infectious viremia [13]. Lorenzi *et al*. found identical *env* sequences from independent VOA cultures and proviruses that were detected longitudinally over a 4–6 month interval in four participants, suggesting the persistence of HIV-infected clones [14]. However, this study was limited by the small number of participants (N = 4) and the use of short amplicons (~2.9 kb) that limit the certainty of clonality [37].

To probe for replication-competent clones, we searched for identical sequences between replication-competent viruses recovered from the VOA, HIV DNA, and/or cell-associated HIV RNA in samples from eight donors on long-term suppressive ART who had outgrowth virus that was fully susceptible to their current ART drug regimen. Identical near full-length sequence matches were recovered among multiple VOA wells for six of eight donors. Given the high proviral sequence diversity for all donors, the likelihood of detecting identical near full-length sequences among two VOA wells in the absence of clonal expansion was calculated using the Poisson probability distribution function as P <10^−23^. In several instances, we found identical sequences in more than two different VOA wells (up to six), which is an even less probable event. These findings strongly suggest that cells harboring replication-competent proviruses underwent clonal expansion *in vivo*. One of the other two donors had identical p6-PR-RT sequences from one VOA well and one cell-associated HIV RNA molecule, suggesting that this donor had expanded cell clones carrying replication-competent HIV proviruses. However, near full-length sequence matches between VOA wells and HIV proviruses or cell-associated HIV RNA would provide stronger evidence. The final donor did not have any recovered viral sequence matches.

A median 57% of the isolated replication-competent viruses had identical p6-PR-RT sequence matches in other VOA wells, HIV DNA, or cell-associated HIV RNA, suggesting that a large fraction of the inducible, replication-competent HIV reservoir consists of CD4^+^ T-cells carrying identical HIV sequences. By contrast, proviral sequences matching the replicating viral sequences were often undetected, indicating that only a small fraction of infected CD4^+^ T-cells carry replication-competent proviruses, as has been reported by others [15].

For all donors, multiple identical p6-PR-RT sequences were isolated from HIV DNA and/or cell-associated HIV RNA that did not match VOA sequences. One likely explanation for this observation is that cells carrying defective proviruses also underwent clonal expansion *in vivo* [10–12, 24, 25, 27], although it is also possible that these identical proviruses were intact but were not induced in the VOA [24, 38].

One potential explanation for detecting identical near full-length sequences from multiple VOA wells is ongoing *in vivo* HIV replication. However, there are several convincing arguments against this explanation. Firstly, all donors in this study were on long-term ART and had suppressed viremia without “blips” for longer than four years (Table 1). Secondly, ongoing viral replication would be expected to produce viral evolution due to the extensive error rate of the viral replication cycle [39] and likely lead to the emergence of drug resistance mutations. The absence of drug resistance mutations in near full-length viral sequences from multiple p24-positive VOA wells is not consistent with ongoing viral replication *in vivo*. Finally, other studies have not shown evidence of ongoing viral replication in donors during appropriate long-term ART, as discussed above [8, 9, 32–35].

Another possible explanation for observing identical sequences from different VOA wells is that a single dominant virus established latency. Although it is theoretically possible that two identical proviruses arose from independent infections of cells with identical viral genomes and persisted long-term, this is improbable given the short life span of the vast majority of infected cells [40], the well-described error rate of reverse transcription during viral replication [28, 29, 41, 42], and the high proviral diversity in infected cells that persisted on ART. Each donor had high proviral sequence diversity (APD 0.5–2.2%, Table 2) and, more importantly, the different groups of identical sequences obtained from different VOA wells within each donor were genetically diverse (APD 0.5–2.1%, median 16 bp differences [range 7–24] between 1.3 kb p6-PR-RT VOA sequences). In the context of these diverse proviral populations, the probability of observing identical sequences from different VOA wells if clonal expansion had not occurred was calculated to be <10^−23^. Taken together, these findings strongly suggest that the identical sequences observed across VOA wells did not arise from infection of cells with genetically identical virions but rather from clonal expansion.

Integration site analysis with linkage to full-length proviral sequences would provide additional proof that an HIV-infected cell with a replication-competent provirus has undergone clonal expansion [13]. However, current procedures for integration site analysis are labor intensive and have insufficient sensitivity to detect the rare (average <1%), clonally expanded replication-competent proviruses that we detected by VOA. The limited sensitivity of such assays may explain why studies using these assays have produced conflicting results. In particular, replication-competent clones could be identified in a patient with a massively expanded clone that constituted ~13% of all proviruses [13] but could not be identified in 75 clones from eight other donors [12]. Despite the limitation of integration site assays, our data showing identical, near full-length viral sequences from different VOA wells is very unlikely to be observed by chance sampling without clonal expansion (P <10^−23^), and thus strongly suggest clonal expansion of infected cells carrying replication-competent proviruses.

Cells carrying intact proviruses may undergo clonal expansion through multiple different mechanisms. Homeostatic proliferation may contribute to the proliferation of infected cells [43]. Integrations in cancer-associated genes are overrepresented in clonal expansions of cells and increase in proportion over time *in vivo*, indicating that HIV integration sites can influence the proliferation and survival of cells [10, 11]. Simonetti *et al*. observed robust expansion of an infectious clone *in vivo* that likely arose from persistent stimulation by tumor antigen [13]. A recent study has shown that *ex vivo* T-cell activation by PMA and ionomycin can lead to the proliferation of clones with intact proviruses [38]. Thus, stimulation by antigen or mitogen are potential mechanisms for clonal expansion of cells with intact proviruses.

In summary, we provide strong evidence that a large proportion (>50%) of the inducible, replication-competent HIV reservoir consists of CD4^+^ T-cells carrying identical proviral sequences. Our findings suggest that clonal expansion is an important mechanism for the persistence of the HIV reservoir during suppressive ART and challenges the notion of a stable latent reservoir that consists only of long-lived infected cells from original infection events. HIV cure efforts will likely need to target an HIV reservoir composed, at least in part, of proliferating infected cells.

## Methods

### Study subjects

Eligible donors included HIV-1-positive adults ≥ 18 years old who were on suppressive ART (plasma HIV RNA < 50 copies/mL) for > 2 years and whose outgrowth viruses did not have resistance mutations that would reduce susceptibility to their ART regimen. Eight subjects were recruited as a convenience cohort from the University of Pittsburgh AIDS Center for Treatment. All study subjects provided written informed consent. Clinical specimen donation protocols were approved by the University of Pittsburgh Institutional Review Board, PRO13070189 and PRO14120068.

### Isolation of PBMC, total CD4^+^ T-cells, and resting CD4^+^ T-cells

Large-volume phlebotomy (180–360 mL whole blood) was performed on all donors. PBMC were isolated by Ficoll-Paque density gradient centrifugation. Total CD4^+^ T-cells were isolated from PBMC by negative selection using the EasySep Human CD4+ T-cell Enrichment Kit (STEMCELL). Resting CD4^+^ T-cells were isolated from PBMC by negative selection using the EasySep Human Custom Enrichment Kit (STEMCELL) that depletes cells expressing CD8, CD14, CD16, CD19, CD20, CD36, CD56, CD66b, CD123, TCRγ/δ, glycophorin A, CD69, CD25, and HLA-DR. Fresh total and/or resting CD4^+^ T-cells were used for the quantitative viral outgrowth assay. Aliquots of 1x10^6^ cells of each cell type were also cryopreserved at -80°C for subsequent nucleic acid analysis.

### Quantification of plasma viral load

Plasma was harvested by initial centrifugation of whole blood at 400 x g for 10 min. Plasma debris was removed by additional centrifugation at 1350 x g for 15 min. Cell-free plasma was stored at -80°C. The plasma viral load was quantified from thawed plasma samples using a qRT-PCR Single-Copy Assay (SCA) with primers that target a conserved region encoding integrase, as previously described [16].

### Quantitative viral outgrowth assay (VOA)

VOAs were performed using total and/or resting CD4^+^ T-cells as previously described [19]. Each VOA used 6 million to 13 million CD4^+^ T-cells seeded in 3-fold serial dilutions with six replicates per dilution, beginning with 1 million cells per well. Wells that were positive for p24 by ELISA (Alliance HIV-1 P24 Antigen ELISA Kit, Perkin Elmer) on days 14 or 21 had their supernatants harvested and stored at -80°C for subsequent single-genome sequencing. Maximum likelihood estimates were applied to calculate infectious units per million cells (IUPM) [44].

### Quantification of cell-associated HIV unspliced RNA and total HIV DNA

Cell-associated HIV unspliced RNA and total HIV DNA were amplified from cryopreserved aliquots of 3 x 10^4^ to 1 x 10^6^ total CD4^+^ T-cells using qRT-PCR and qPCR, respectively, as previously described [18]. HIV copies were normalized to cell number that was quantified by qPCR targeting *CCR5*, as previously described [18].

### Single-genome sequencing (SGS) of p6-PR-RT from p24-positive VOA wells and from HIV DNA and cell-associated RNA in uncultured blood mononuclear cells

SGS targeting a region encoding p6 of gag, protease, and the first ~900 nucleotides of reverse transcriptase (p6-PR-RT, nt 1893–3408; HXB2 positions [20]) was performed on HIV RNA from cryopreserved p24-positive VOA supernatants and cell-associated HIV DNA and/or RNA from cryopreserved uncultured blood mononuclear cells. Extraction of nucleic acids from VOA supernatants was performed as previously described [21, 22], except with initial centrifugation of the supernatant at 5,300 x g for 10 min at 4°C. cDNA was synthesized using the SuperScript III First-Strand Synthesis System (Invitrogen), with each reaction containing 40 μL of supernatant extract, 5 μL of 10 mM deoxynucleotide triphosphates, and 5 μL of 2 μM reverse primer targeting the RT-coding region (5’-CTATTAAGTATTTTGATGGGTCATAA-3’). After denaturation at 65°C for 10 min, samples were quenched on ice followed by the addition of 10 μL of 10X RT buffer, 20 μL of 25 mM MgCl_2_, 1 μL of DTT, 17.5 μL of molecular-grade water, 1 μL of RNase-Out, and 0.5 μL of SuperScript III RT. Next, samples were incubated at 45°C for 50 min, 85°C for 10 min, and then at 4°C.

To perform SGS on HIV DNA and cell-associated HIV RNA, nucleic acid was extracted from PBMC, total CD4^+^ T-cells, or resting CD4^+^ T-cells as previously described [18], with minor modifications. The sonication step was not performed to ensure the integrity of the nucleic acid target for amplification and sequencing. In addition, cell-associated RNA was extracted in aliquots ranging from 3 x 10^4^ to 1 x 10^6^ cells per aliquot. cDNA synthesis from cell-associated HIV RNA was performed as previously described [7, 45].

SGS was performed on the cDNA or DNA cellular extract at a dilution that yielded <30% positive PCR reactions, as previously described [21, 22]. The nested PCR amplicon was ~1.5 kb spanning p6-PR-RT. The PCR product was treated with 20 U of Exonuclease I (Affymetrix) and 4 U of Shrimp Alkaline Phosphatase (Affymetrix). Sequencing was performed using four sequencing primers: 5’-TGTTGGAAATGTGGAAAGGAAGGAC-3’, 5’-ATGGCCCAAAAGTTAAACAATGGC-3’, 5’-TTCTTCTGTCAATGGCCATTGTTTAAC-3’, 5’-TTGCCCAATTCAATTTTCCCACTAA-3’. Sequences were aligned and quality-checked in Sequencher 5.3. Sequences with evidence of multiple templates were excluded from analysis. At least 100 HIV DNA SGS sequences were obtained from each donor. Hypermutant sequences were identified using the Los Alamos Hypermut tool [46]. Neighbor-joining distance analysis was performed in MEGA6. The average pairwise distances (APDs) were calculated in MEGA6 from p6-PR-RT proviral single-genome sequences after exclusion of hypermutant sequences.

### SGS of overlapping half-genomes from virion-associated HIV RNA

VOA p24-positive wells with p6-PR-RT sequences that matched other p24-positive wells underwent overlapping half-genome SGS, with modifications to a previously described method [23]. Viral RNA extraction from cell-free supernatants was performed using the QIAmp Viral RNA Mini Kit (QIAGEN) [23]. cDNA synthesis was performed using the SuperScript III First-Strand Synthesis System (Invitrogen). Separate RT reactions were used to amplify 5’ and 3’ half-genome fragments using the reverse primers listed in S3 Table. First, 30 μL of RNA extract was incubated with 0.25 μM of primer and 0.5 mM dNTP at 65°C for 5 min, with subsequent incubation on ice for 1 min. The cDNA reaction mixture was prepared and added as described [23]; however, 5 U/μL instead of 10 U/μL of SuperScript III was used. The cDNA reactions were carried out at 50°C for 1 h then 55°C for 1 h before heat inactivation of SuperScript III, RNase H treatment, and storage as previously described [23].

Next, SGS of the half-genome cDNA was performed as previously described [23], but with 10 μL reactions and the primers listed in S3 Table. The first-round PCR was performed with 1 μL of diluted cDNA and the second-round PCR was performed with 1 μL of first-round PCR product. PCR-positive wells were identified by agarose gel electrophoresis.

Enzymatic PCR cleanup was performed on PCR-positive wells by incubation with 0.5 U of Exonuclease I (New England Biolabs) and 0.25 U of Shrimp Alkaline Phosphatase (Affymetrix) per PCR reaction at 37°C for 45 min, then 95°C for 5 min. Each well was diluted with H_2_O to a total volume of 120 μL. Sequencing reactions were prepared using 3 μL of cleaned up PCR product, 3 μL of 1.1 μM sequencing primer (S3 Table), and 0.25 μL of BigDye Terminator Ready Reaction Mix (Applied Biosystems) in 1X BigDye Terminator Sequencing Buffer (Applied Biosystems). Cycle sequencing conditions were 96°C for 2 min, 30 cycles of 96°C for 10 s, 50°C for 5 s, 60°C for 4 min, and hold at 4°C.

Extension products were purified using ethanol / EDTA / sodium acetate precipitation. The extension products were mixed with 1 μL of 125 mM EDTA, 1 μL of 3 M sodium acetate, and 25 μL of 100% ethanol per reaction and incubated at room temperature for 30 min. Next, the extension products were collected by centrifugation at 4,122 x g for 30 min at 4°C, decanting, and centrifugation in an inverted orientation at 58 x g for 1 min. Each reaction was then resuspended in 50 μL of 70% ethanol followed by repeat centrifugation, decanting, and inverted centrifugation as described above. Lastly, the extension products were resuspended in 15 μL of HiDye formamide (Life Technologies) per reaction and sequenced using an ABI 3730XL.

Amplicons were aligned and analyzed in Sequencher 5.3 and MEGA6. For each VOA well, near full-length consensus sequences of HIV RNA (nt 582–9606; HXB2 positions [20]) were assembled from the consensus of overlapping 5’ and 3’ half-genome SGS sequences, with different viral variants determined by pairing half-genomes with identical overlapping genomes. For each viral variant, the half-genome sequences did not differ by more than is expected from errors introduced during cDNA synthesis and PCR [21]. Near full-length genome sequences were also analyzed for drug resistance mutations using the Stanford University HIV Drug Resistance Database [47]. Recombination was assessed using SimPlot [30]. Coreceptor tropism was determined using Geno2Pheno bioinformatics software [31].

### Statistical analysis to calculate the probability of observing identical, near full-length HIV RNA sequences across VOA wells

The probability of observing the observed numbers of identical, near full-length, HIV RNA sequences across VOA wells if clonal expansion had not occurred was calculated for each donor. The number of replication-competent, proviral sequences that were assayed was calculated as the mathematical product of the number of CD4^+^ T-cells assayed in the VOA, the frequency of infected cells in CD4^+^ T-cells, and the frequency of infected cells that are expected to contain intact proviruses (2%) [27]. The APD of intact proviruses was estimated from p6-PR-RT proviral single-genome sequences after exclusion of hypermutant sequences. The probability of observing an identical pair of sequences between VOA wells by chance if clonal expansion had not occurred was calculated by a Poisson distribution using the APD and the length of the near full-length HIV amplicons. This probability multiplied by the total number of possible sequence pairs yields the expected number of identical sequence pairs to be observed. Because this expected number was less than one for all donors, a Poisson probability distribution function was used to determine the probability of observing the number of observed identical sequence pairs or greater if clonal expansion had not occurred.

## Supporting information

S1 Fig Near Full-Length Sequencing for Donor 1. *(a)* Single-genome sequencing was used to amplify and sequence 5’ and 3’ overlapping half-genomes from single viral templates from p24-positive viral outgrowth cultures. The 5’ and 3’ half-genome sequences were linked using their overlapping genomic region (▪▪▪). *(b)* Consensus near full-length viral sequences were constructed from linked half-genome sequences. These near full-length sequences were compared to each other and to p6-PR-RT sequence matches to verify their identity.
(TIF)

S2 Fig Neighbor-joining distance tree of sequences in p24-positive viral outgrowth assay wells and in HIV DNA sequences from blood mononuclear cells (Donor 2). The tree was constructed as described in Fig 2. Identical p6-PR-RT sequences were recovered from two p24-positive viral outgrowth assay (VOA) wells and one provirus (blue arrow), with confirmed matches of viral RNA in VOA wells by overlapping half-genome sequencing (*). A second set of identical p6-PR-RT sequences was recovered from four p24-positive VOA wells (red arrow), with confirmed matches of viral RNA by overlapping half-genome sequencing (*) among the VOA wells except for one VOA well (orange closed diamond). Near full-length matching sequences obtained from the VOA wells appeared intact without large deletions, frame-shift mutations, or disabling stop codons.
(TIF)

S3 Fig Neighbor-joining distance tree of sequences in p24-positive viral outgrowth assay wells and in HIV DNA sequences from blood mononuclear cells (Donor 3). The tree was constructed as described in Fig 2. Identical p6-PR-RT sequences were recovered from one p24-positive viral outgrowth assay (VOA) well and two proviruses (pink arrow). A second set of identical p6-PR-RT sequences was recovered from two p24-positive VOA wells (red arrow), with confirmed matches of viral RNA by overlapping half-genome sequencing (*). A third set of identical p6-PR-RT sequences was recovered from a single p24-positive VOA well and four proviruses (blue arrow). Recovered near full-length matching sequences from the VOA wells appeared intact without large deletions, frame-shift mutations, or disabling stop codons.
(TIF)

S4 Fig Neighbor-joining distance tree of sequences in p24-positive viral outgrowth assay wells and in HIV DNA sequences from blood mononuclear cells (Donor 4). The tree was constructed as described in Fig 2. Identical p6-PR-RT sequences were recovered from one p24-positive viral outgrowth assay (VOA) well and one provirus (red arrow). Identical p6-PR-RT sequences were recovered from four p24-positive VOA wells (blue arrow), with confirmed matches of viral RNA by overlapping half-genome sequencing (*) among the VOA wells except for one VOA well (green closed diamond). Near full-length matching sequences obtained from the VOA wells appeared intact without large deletions, frame-shift mutations, or disabling stop codons.
(TIF)

S5 Fig Neighbor-joining distance tree of sequences in p24-positive viral outgrowth assay wells and of HIV DNA sequences from blood mononuclear cells (Donor 5). The tree was constructed as described in Fig 2. No identical p6-PR-RT sequences were found across viral outgrowth assay (VOA) wells or between VOA wells and HIV DNA.
(TIF)

S6 Fig Neighbor-joining distance tree of sequences in p24-positive viral outgrowth assay wells and in HIV DNA sequences from blood mononuclear cells (Donor 6). The tree was constructed as described in Fig 2. Identical p6-PR-RT sequences were recovered from a single p24-positive viral outgrowth assay (VOA) well and two proviruses (blue arrow). A second set of identical p6-PR-RT sequences was recovered from two p24-positive VOA wells (red arrow), with confirmed matches of viral RNA by overlapping half-genome sequencing (*). Near full-length matching sequences obtained from the VOA wells appeared intact without large deletions, frame-shift mutations, or disabling stop codons.
(TIF)

S7 Fig Neighbor-joining distance tree of sequences in p24-positive viral outgrowth assay wells and in HIV DNA and RNA from blood mononuclear cells (Donor 7). p6-PR-RT single-genome sequences were obtained from HIV DNA and cell-associated HIV RNA from independent PBMC extractions. Black circles represent HIV DNA sequences. Different colored square and triangle symbols represent HIV RNA sequences from different PBMC extractions. p6-PR-RT single-genome sequences were also obtained from a p24-positive well from a viral outgrowth assay (VOA) performed using resting CD4^+^ T-cells. Identical p6-PR-RT sequences were recovered from the p24-positive VOA well and one cell-associated HIV RNA molecule (blue arrow). The near full-length consensus sequence from the VOA well appeared intact without large deletions, frame-shift mutations, or disabling stop codons.
(TIF)

S8 Fig Neighbor-joining distance tree of sequences in p24-positive viral outgrowth assay wells and in HIV DNA sequences from blood mononuclear cells (Donor 8). The tree was constructed as described in Fig 2. p6-PR-RT single-genome sequences were obtained from HIV DNA in PBMC. p6-PR-RT single-genome sequences were obtained from independent, p24-positive viral outgrowth assay (VOA) wells performed using resting CD4^+^ T-cells from an initial time point and from total CD4^+^ T-cells at a different time point 18 months later. Identical p6-PR-RT sequences were recovered from two p24-positive VOA wells from the two different time points (red arrow), with confirmed matches of viral RNA by overlapping half-genome sequencing (*). Identical p6-PR-RT sequences were also recovered from three other pairs of p24-positive VOA wells from two different time points (blue, pink, and green arrows). Identical p6-PR-RT sequences were also recovered from two p24-positive VOA wells from the same time point (teal arrow). Near full-length matching sequences obtained from the VOA wells appeared intact without large deletions, frame-shift mutations, or disabling stop codons.
(TIF)

S1 Table HIV drug resistance and coreceptor tropism analysis of sequences from p24-positive viral outgrowth assay wells with identical sequence matches. Drug resistance mutations were identified using the Stanford University HIV Drug Resistance Database. Coreceptor tropism was predicted by Geno2pheno for X4 tropism.
(DOCX)

S2 Table Frequencies of identical sequence matches between HIV DNA and p24-positive viral outgrowth assay wells. The frequencies of p6-PR-RT sequence matches between HIV DNA sequences in uncultured PBMC and virion-associated HIV RNA sequences in p24-positive viral outgrowth assay (VOA) were calculated. Hypermutant HIV DNA sequences with hypermutant signatures were excluded from this analysis.
(DOCX)

S3 Table Half-genome single-genome sequencing primers. (DOCX)
