# Supplementary figures and images for: Proviruses with identical sequences comprise a large fraction of the replication-competent HIV reservoir
Source: PLoS Pathog. 2017 Mar 22;13(3):e1006283. doi: 10.1371/journal.ppat.1006283 (PMC5378418; doi:10.1371/journal.ppat.1006283)

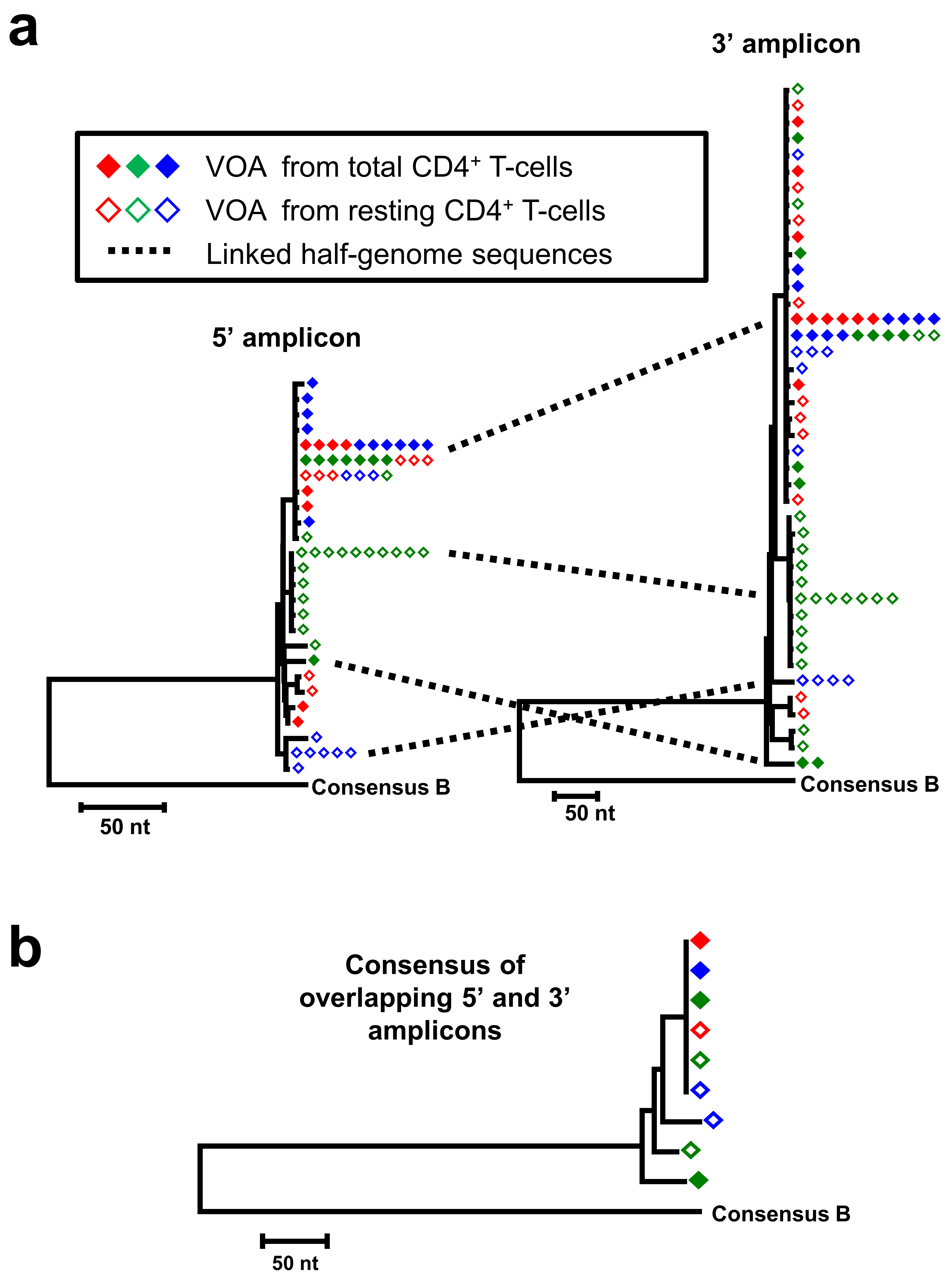

Supplement: S1 Fig — (a) Single-genome sequencing was used to amplify and sequence 5’ and 3’ overlapping half-genomes from single viral templates from p24-positive viral outgrowth cultures. The 5’ and 3’ half-genome sequences were linked using their overlapping genomic region (▪▪▪). (b) Consensus near full-length viral sequences were constructed from linked half-genome sequences. These near full-length sequences were compared to each other and to p6-PR-RT sequence matches to verify their identity. (TIF) [file ppat.1006283.s001.TIF]

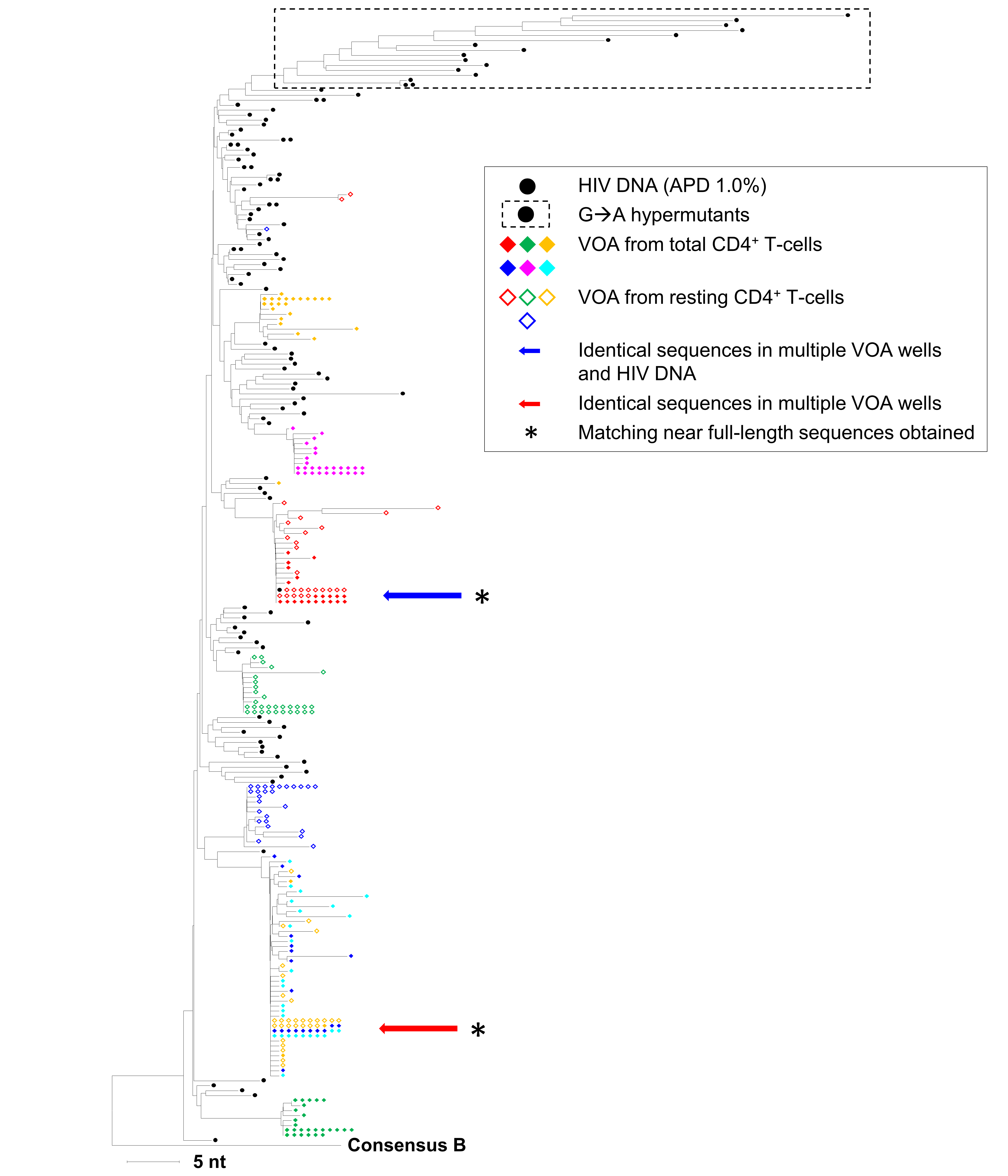

Supplement: S2 Fig — The tree was constructed as described in Fig 2. Identical p6-PR-RT sequences were recovered from two p24-positive viral outgrowth assay (VOA) wells and one provirus (blue arrow), with confirmed matches of viral RNA in VOA wells by overlapping half-genome sequencing (*). A second set of identical p6-PR-RT sequences was recovered from four p24-positive VOA wells (red arrow), with confirmed matches of viral RNA by overlapping half-genome sequencing (*) among the VOA wells except for one VOA well (orange closed diamond). Near full-length matching sequences obtained from the VOA wells appeared intact without large deletions, frame-shift mutations, or disabling stop codons. (TIF) [file ppat.1006283.s002.TIF]

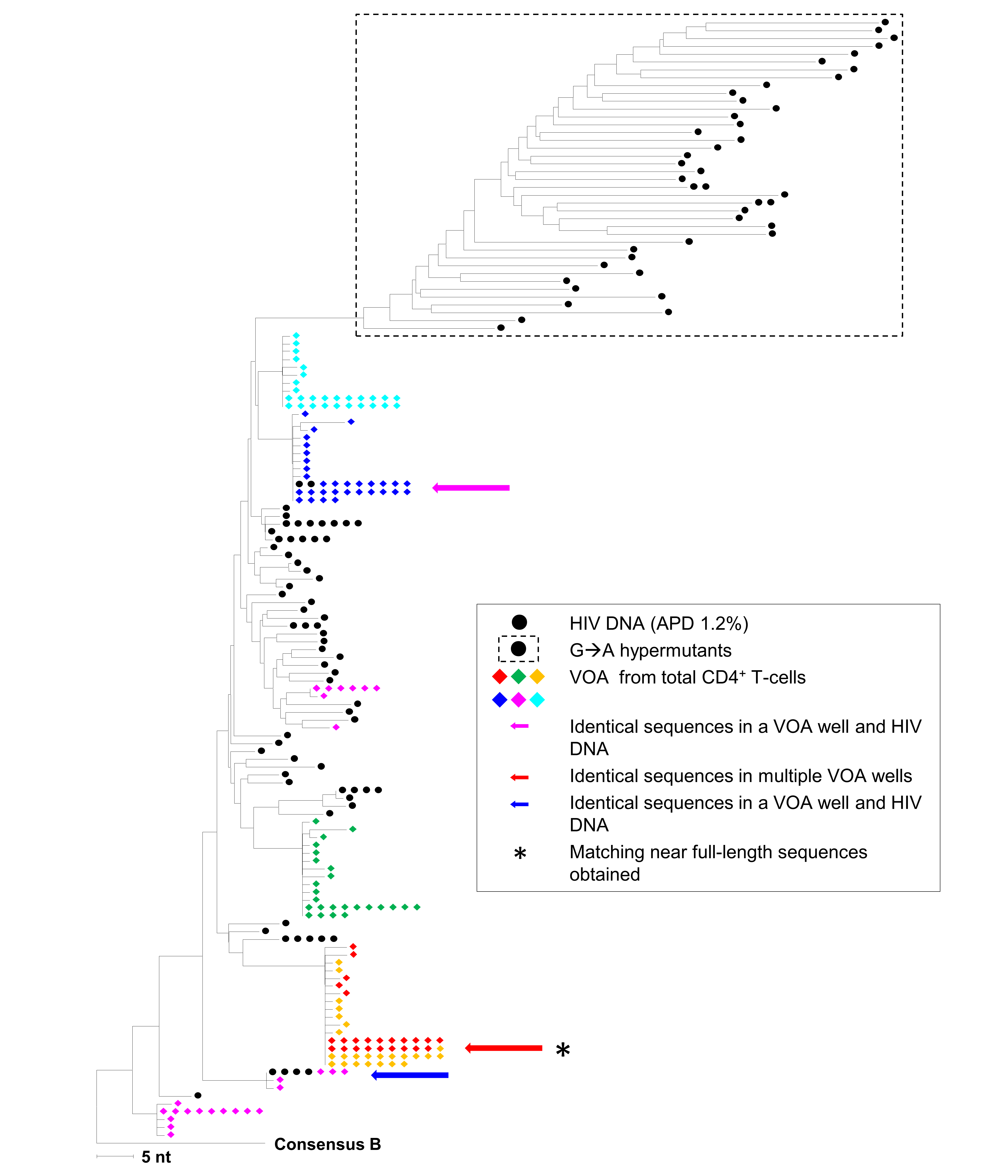

Supplement: S3 Fig — The tree was constructed as described in Fig 2. Identical p6-PR-RT sequences were recovered from one p24-positive viral outgrowth assay (VOA) well and two proviruses (pink arrow). A second set of identical p6-PR-RT sequences was recovered from two p24-positive VOA wells (red arrow), with confirmed matches of viral RNA by overlapping half-genome sequencing (*). A third set of identical p6-PR-RT sequences was recovered from a single p24-positive VOA well and four proviruses (blue arrow). Recovered near full-length matching sequences from the VOA wells appeared intact without large deletions, frame-shift mutations, or disabling stop codons. (TIF) [file ppat.1006283.s003.TIF]

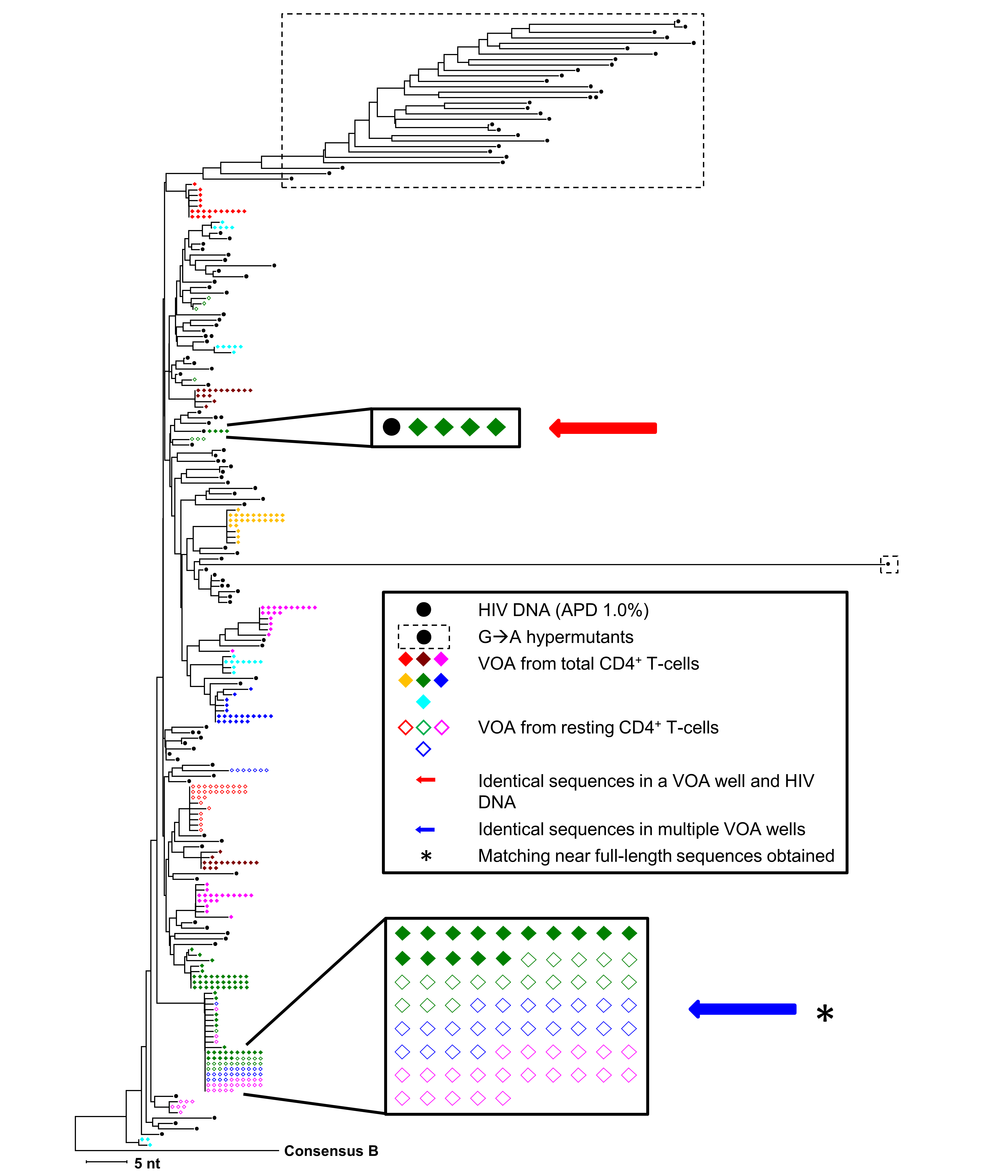

Supplement: S4 Fig — The tree was constructed as described in Fig 2. Identical p6-PR-RT sequences were recovered from one p24-positive viral outgrowth assay (VOA) well and one provirus (red arrow). Identical p6-PR-RT sequences were recovered from four p24-positive VOA wells (blue arrow), with confirmed matches of viral RNA by overlapping half-genome sequencing (*) among the VOA wells except for one VOA well (green closed diamond). Near full-length matching sequences obtained from the VOA wells appeared intact without large deletions, frame-shift mutations, or disabling stop codons. (TIF) [file ppat.1006283.s004.TIF]

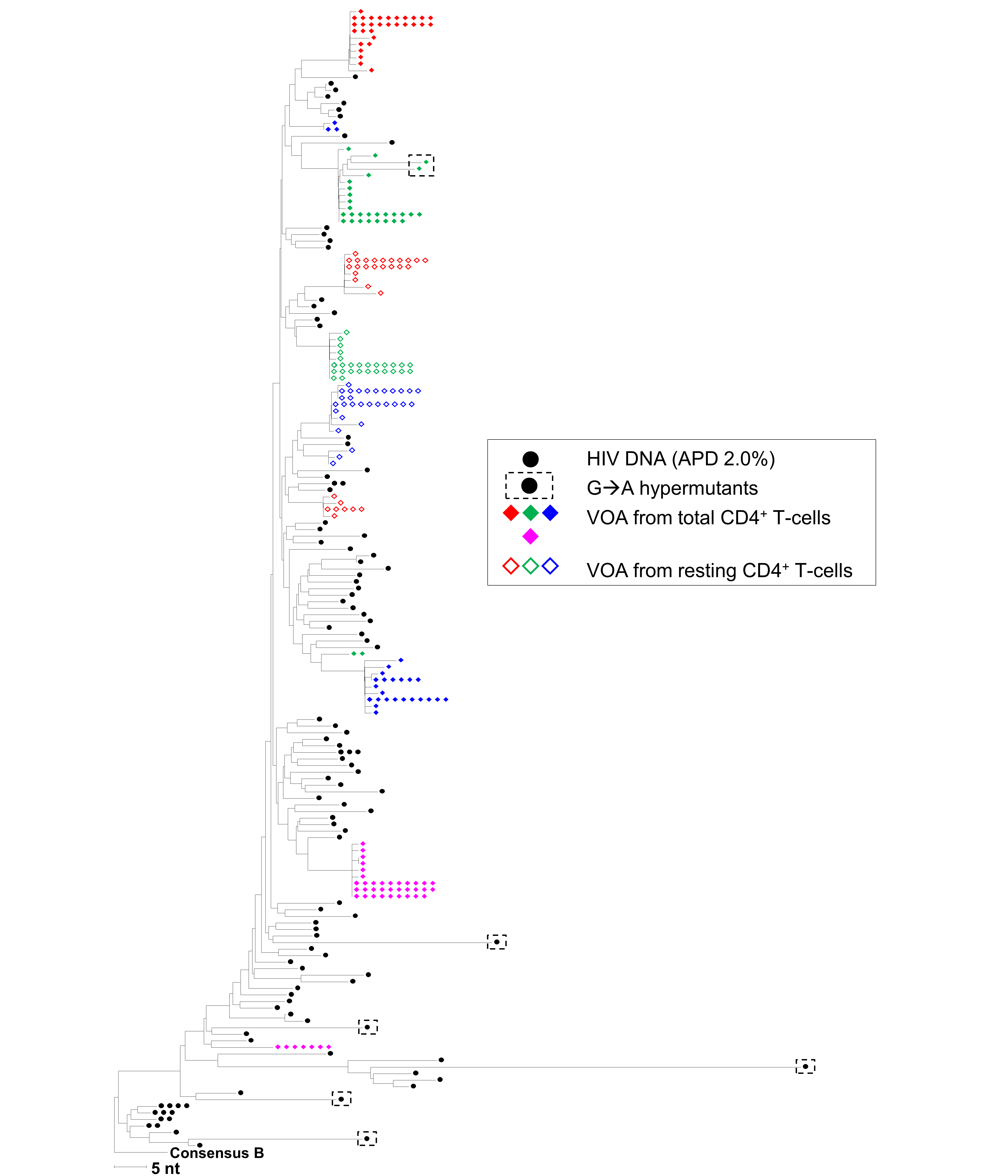

Supplement: S5 Fig — The tree was constructed as described in Fig 2. No identical p6-PR-RT sequences were found across viral outgrowth assay (VOA) wells or between VOA wells and HIV DNA. (TIF) [file ppat.1006283.s005.TIF]

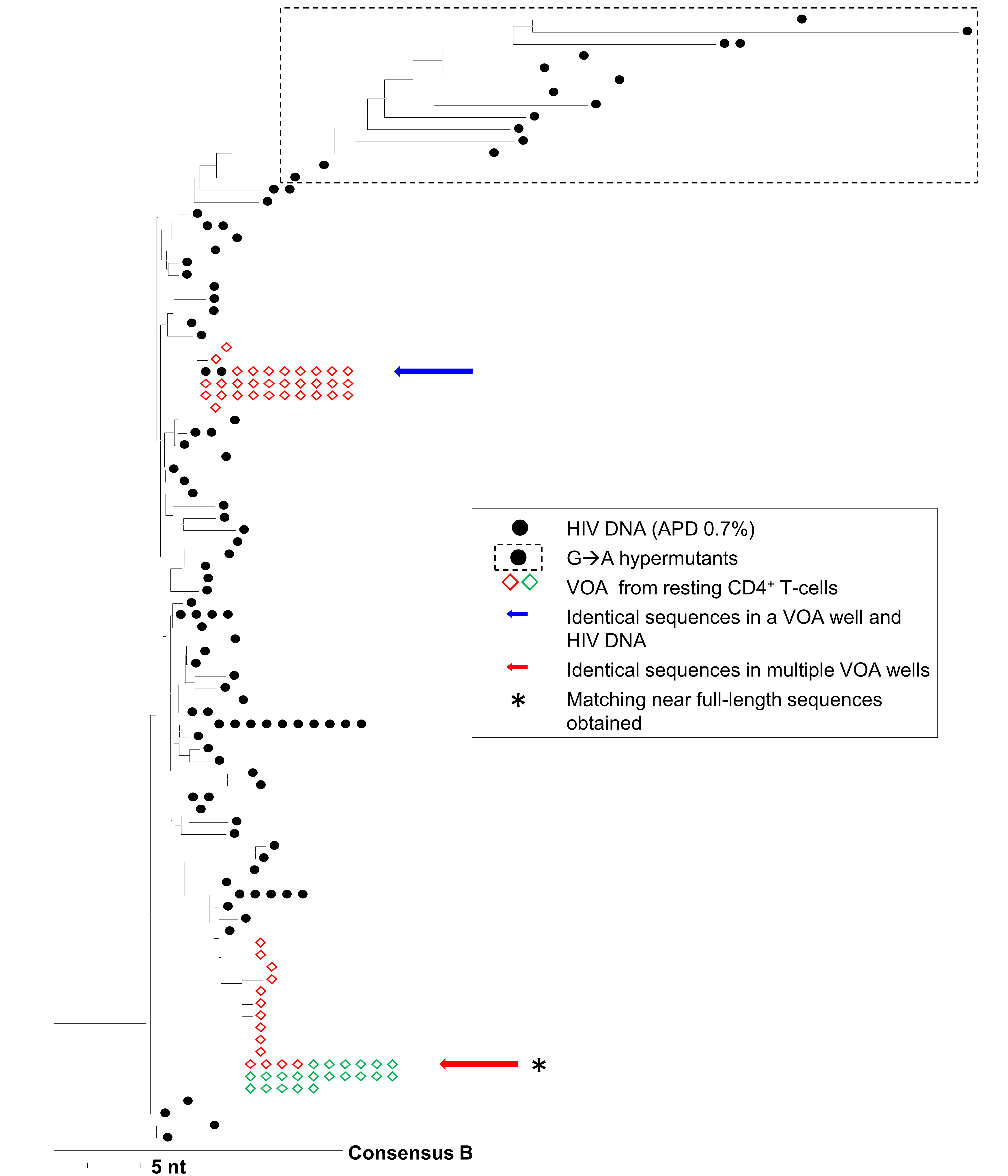

Supplement: S6 Fig — The tree was constructed as described in Fig 2. Identical p6-PR-RT sequences were recovered from a single p24-positive viral outgrowth assay (VOA) well and two proviruses (blue arrow). A second set of identical p6-PR-RT sequences was recovered from two p24-positive VOA wells (red arrow), with confirmed matches of viral RNA by overlapping half-genome sequencing (*). Near full-length matching sequences obtained from the VOA wells appeared intact without large deletions, frame-shift mutations, or disabling stop codons. (TIF) [file ppat.1006283.s006.TIF]

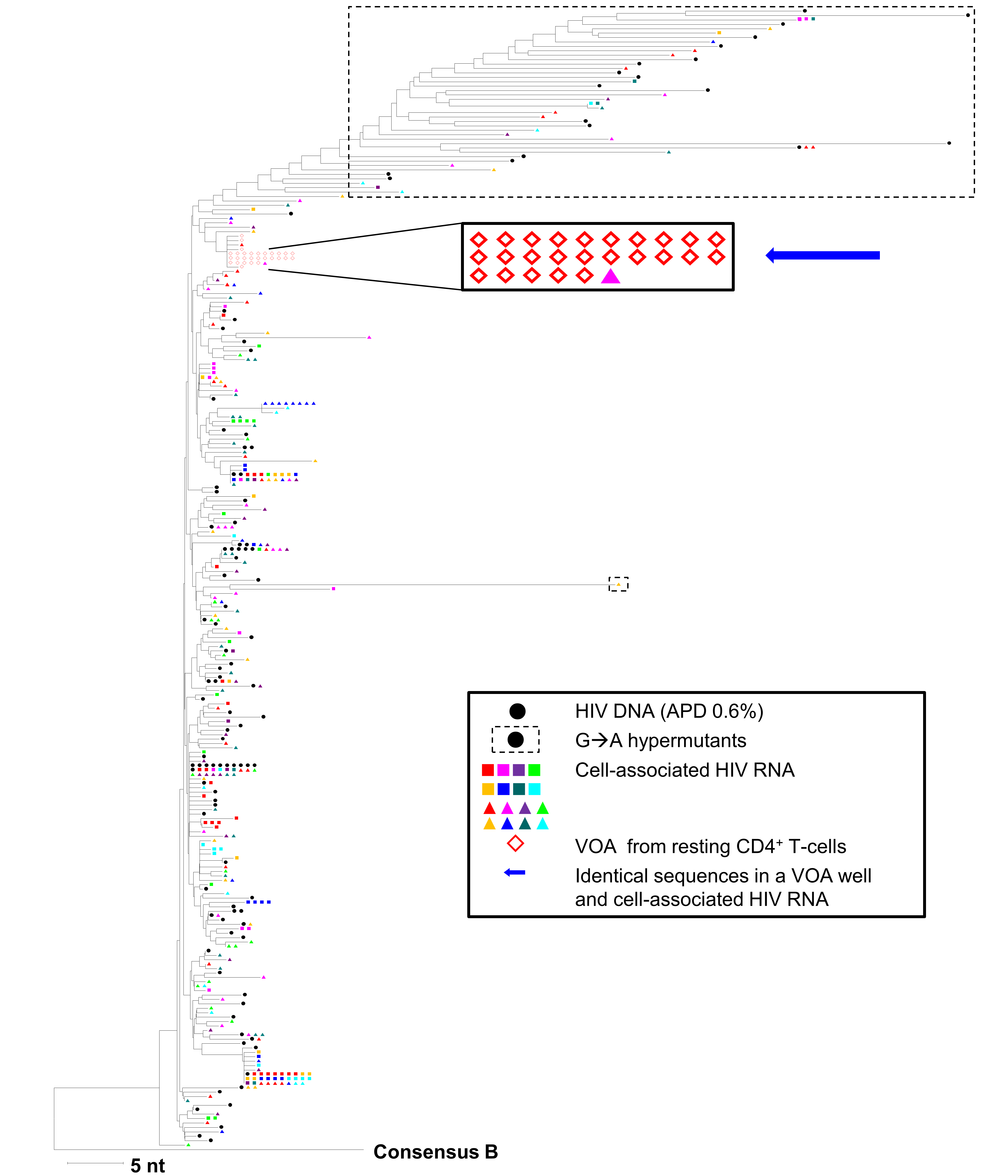

Supplement: S7 Fig — p6-PR-RT single-genome sequences were obtained from HIV DNA and cell-associated HIV RNA from independent PBMC extractions. Black circles represent HIV DNA sequences. Different colored square and triangle symbols represent HIV RNA sequences from different PBMC extractions. p6-PR-RT single-genome sequences were also obtained from a p24-positive well from a viral outgrowth assay (VOA) performed using resting CD4+ T-cells. Identical p6-PR-RT sequences were recovered from the p24-positive VOA well and one cell-associated HIV RNA molecule (blue arrow). The near full-length consensus sequence from the VOA well appeared intact without large deletions, frame-shift mutations, or disabling stop codons. (TIF) [file ppat.1006283.s007.TIF]

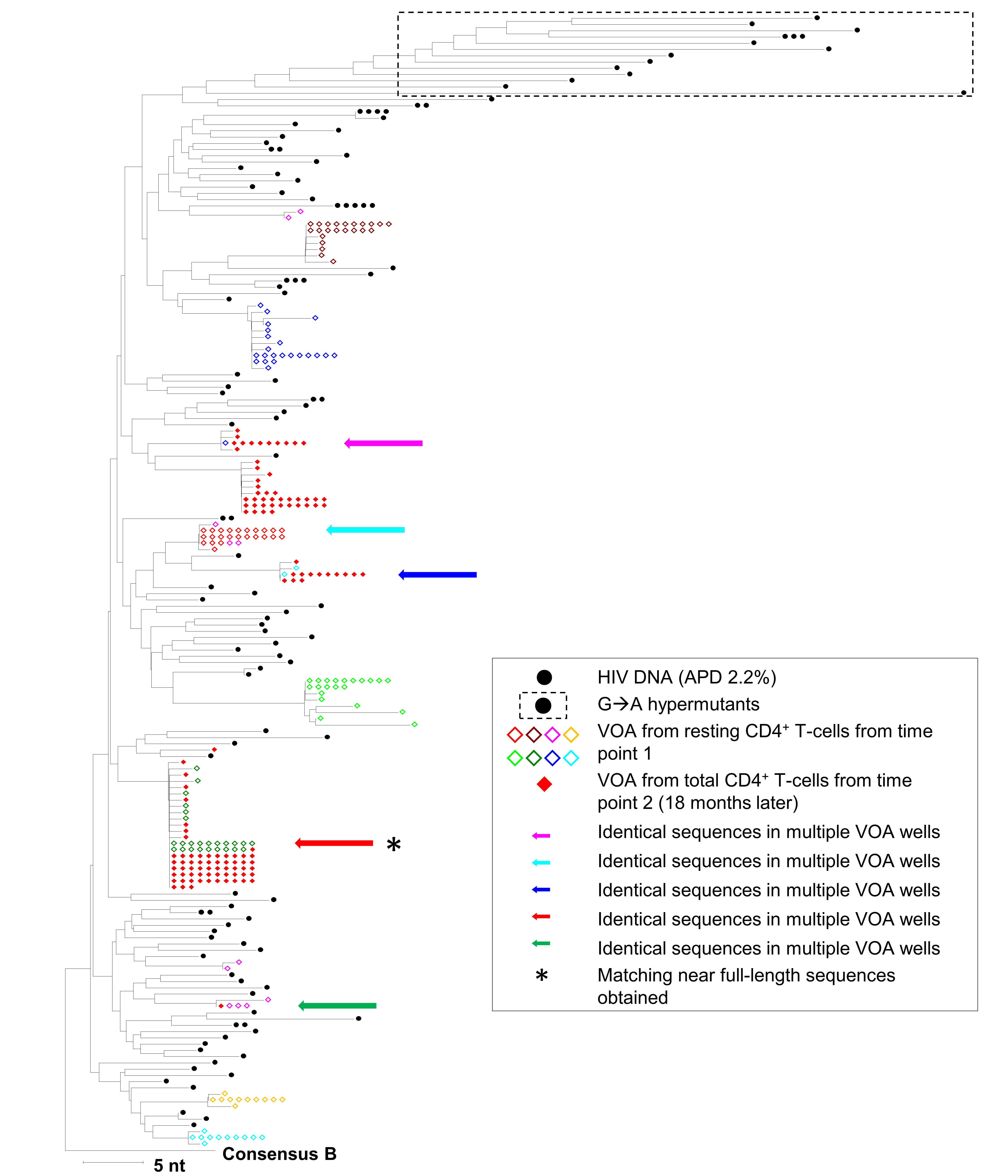

Supplement: S8 Fig — The tree was constructed as described in Fig 2. p6-PR-RT single-genome sequences were obtained from HIV DNA in PBMC. p6-PR-RT single-genome sequences were obtained from independent, p24-positive viral outgrowth assay (VOA) wells performed using resting CD4+ T-cells from an initial time point and from total CD4+ T-cells at a different time point 18 months later. Identical p6-PR-RT sequences were recovered from two p24-positive VOA wells from the two different time points (red arrow), with confirmed matches of viral RNA by overlapping half-genome sequencing (*). Identical p6-PR-RT sequences were also recovered from three other pairs of p24-positive VOA wells from two different time points (blue, pink, and green arrows). Identical p6-PR-RT sequences were also recovered from two p24-positive VOA wells from the same time point (teal arrow). Near full-length matching sequences obtained from the VOA wells appeared intact without large deletions, frame-shift mutations, or disabling stop codons. (TIF) [file ppat.1006283.s008.TIF]
