# Supplementary material for: Proviruses with identical sequences comprise a large fraction of the replication-competent HIV reservoir
Source: PLoS Pathog. 2017 Mar 22;13(3):e1006283. doi: 10.1371/journal.ppat.1006283 (PMC5378418; doi:10.1371/journal.ppat.1006283)
Supplement: S1 Table — Drug resistance mutations were identified using the Stanford University HIV Drug Resistance Database. Coreceptor tropism was predicted by Geno2pheno for X4 tropism. (DOCX) [file ppat.1006283.s009.docx]

**S1 Table. HIV drug resistance and coreceptor tropism analysis of sequences from p24-positive viral outgrowth assay wells with identical sequence matches.** Drug resistance mutations were identified using the Stanford University HIV Drug Resistance Database. Coreceptor tropism was predicted by Geno2pheno for X4 tropism.

| Donor ID | ART Regimen | Clones | Coreceptor tropism | Drug Resistance Mutations to Donor’s ART Regimen |
| --- | --- | --- | --- | --- |
| 1 | DRV/r / ETR / RAL | F2, F3, F4, F6, F7.2, F8 | R5-tropic (23.4% FPR) | None |
| 2 | EFV / FTC / TDF | A1, A5 | R5-tropic (42.6% FPR) | None |
|  |  | A3, A4, A6 | R5-tropic (74.6% FPR) | None |
| 3 | EFV / FTC / TDF | F2, F4 | X4-tropic (5.7% FPR) | None |
|  |  | 1.5 | Env region not available in obtained sequences | None |
|  |  | 2A | X4-tropic (0.2% FPR) | None |
| 4 | FTC / TDF / RAL | B2, B3, B4 | X4-tropic (0.5% FPR) | None |
| 5 | DRV/r / FTC / RAL / TDF | No sequence matches observed |  |  |
| 6 | ATV/r / FTC / TDF | B3.1, B4 | R5-tropic (34.6% FPR) | None |
|  |  | B3.2 | R5-tropic (34.6% FPR) | None |
| 7 | EFV / FTC / TDF | S1 | R5-tropic (25% FPR) | None |
| 8 | EFV / FTC / TDF | B8, C2, C4, C6 | R5-tropic (89.1% FPR) | None |
|  |  | C1 | R5-tropic (91% FPR) | None |
|  |  | C3 | R5-tropic (89.1% FPR) | None |
|  |  | B9 | R5-tropic (86.5% FPR) | None |
|  |  | 1M2 | Env region not available in obtained sequences | None |

ATV/r, atazanavir/ritonavir; DRV/r, darunavir/ritonavir; EFV, efavirenz; ETR, etravirine; FTC, emtricitabine; RAL, raltegravir; TDF, tenofovir disoproxil fumarate.

R5, CCR5-tropic; X4, CXCR4-tropic; FPR, false-positive rate for incorrectly identifying the sequence as X4-tropic.
