# Supplementary material for: Proviruses with identical sequences comprise a large fraction of the replication-competent HIV reservoir
Source: PLoS Pathog. 2017 Mar 22;13(3):e1006283. doi: 10.1371/journal.ppat.1006283 (PMC5378418; doi:10.1371/journal.ppat.1006283)
Supplement: S2 Table — The frequencies of p6-PR-RT sequence matches between HIV DNA sequences in uncultured PBMC and virion-associated HIV RNA sequences in p24-positive viral outgrowth assay (VOA) were calculated. Hypermutant HIV DNA sequences with hypermutant signatures were excluded from this analysis. (DOCX) [file ppat.1006283.s010.docx]

**S2 Table. Frequencies of identical sequence matches between HIV DNA and p24-positive viral outgrowth assay wells.** The frequencies of p6-PR-RT sequence matches between HIV DNA sequences in uncultured PBMC and virion-associated HIV RNA sequences in p24-positive wells from the viral outgrowth assay (VOA) were calculated. Hypermutant HIV DNA sequences with hypermutant signatures were excluded from this analysis.

| **Donor ID** | **Number of HIV DNA sequences assayed** | **APD of HIV DNA sequences (%)** | **Number of HIV DNA sequence matches to VOA sequences** | **Frequency of HIV DNA sequence matches to VOA sequences** |
| --- | --- | --- | --- | --- |
| 1 | 107 | 0.5 | 0 | < 0.9% |
| 2 | 98 | 1.0 | 1 | 1.0% |
| 3 | 66 | 1.2 | 4 matches to one IVR sequence | 6.1% |
|  |  |  | 2 matches to another IVR sequence | 3.0% |
| 4 | 83 | 1.0 | 1 | 1.2% |
| 5 | 105 | 2.0 | 0 | < 1.0% |
| 6 | 87 | 0.7 | 2 | 2.3% |
| 7 | 91 | 0.6 | 0 | < 1.1% |
| 8 | 101 | 2.2 | 0 | < 1.0% |
