# Supplementary material for: Proviruses with identical sequences comprise a large fraction of the replication-competent HIV reservoir
Source: PLoS Pathog. 2017 Mar 22;13(3):e1006283. doi: 10.1371/journal.ppat.1006283 (PMC5378418; doi:10.1371/journal.ppat.1006283)
Supplement: S3 Table — (DOCX) [file ppat.1006283.s011.docx]

**S3 Table. Half-genome single-genome sequencing primers.**

| **5’ half-genome cDNA synthesis** | | | |
| --- | --- | --- | --- |
| Primer name | Primer direction | HXB2 position | Primer sequence (5’🡪3’) |
| B5.R1 | Reverse | 5077-5049 | CTTGCCACACAATCATCACCTGCCATCTG |
| **5’ half-genome first-round PCR** | | | |
| Primer name | Primer direction | HXB2 position | Primer sequence (5’🡪3’) |
| B5.F1 | Forward | 538-571 | CCTTGAGTGCTTCAAGTAGTGTGTGCCCGTCTGT |
| B5.R1 | Reverse | 5077-5049 | CTTGCCACACAATCATCACCTGCCATCTG |
| **5’ half-genome second-round PCR** | | | |
| Primer name | Primer direction | HXB2 position | Primer sequence (5’🡪3’) |
| B5.F2 | Forward | 553-581 | GTAGTGTGTGCCCGTCTGTTGTGTGACTC |
| B5.R2 | Reverse | 5068-5040 | CAATCATCACCTGCCATCTGTTTTCCATA |
| **5’ half-genome sequencing primers** | | | |
| Primer name | Primer direction | HXB2 position | Primer sequence (5’🡪3’) |
| B5.F2 | Forward | 553-581 | GTAGTGTGTGCCCGTCTGTTGTGTGACTC |
| B5.R2 | Reverse | 5068-5040 | CAATCATCACCTGCCATCTGTTTTCCATA |
| For1 | Forward | 1061-1082 | GGATAGAGGTAAAAGACACCAA |
| For2 | Forward | 1488-1511 | AAGTGACATAGCAGGAACTACTAG |
| For3 | Forward | 2012-2036 | CTAGGAAAAAGGGCTGTTGGAAATG |
| For4 | Forward | 2385-2410 | AAAATGATAGGGGGAATTGGAGGTTT |
| For5 | Forward | 2869-2893 | CAGTACTAGATGTGGGGGATGCATA |
| For6 | Forward | 3298-3322 | ACAGCTGGACTGTCAATGATATACA |
| For7 | Forward | 3676-3700 | CCACAGAAAGCATAGTAATATGGGG |
| For8 | Forward | 4162-4186 | CACACAAAGGGATTGGAGGAAATGA |
| For9 | Forward | 4535-4558 | AAAATTAGCAGGAAGATGGCCAGT |
| Rev7 | Reverse | 3370-3345 | ATCCCTGGATAAATCTGACTTGCCCA |
| **3’ half-genome cDNA synthesis** | | | |
| Primer name | Primer direction | HXB2 position | Primer sequence (5’🡪3’) |
| B3.R1 | Reverse | 9642-9611 | ACTACTTGAAGCACTCAAGGCAAGCTTTATTG |
| **3’ half-genome first-round PCR (either forward primer used)** | | | |
| Primer name | Primer direction | HXB2 position | Primer sequence (5’🡪3’) |
| B3OL.F1 | Forward | 3676-3700 | CCACAGAAAGCATAGTAATATGGGG |
| HIV.BK3.F1 | Forward | 4749-4771 | ACAGCAGTACAAATGGCAGTATT |
| B3.R1 | Reverse | 9642-9611 | ACTACTTGAAGCACTCAAGGCAAGCTTTATTG |
| **3’ half-genome second-round PCR (either forward primer used)** | | | |
| Primer name | Primer direction | HXB2 position | Primer sequence (5’🡪3’) |
| B3OL.F2 | Forward | 3786-3808 | GAGTGGGAGTTTGTCAATACCCC |
| HIV.BK3.F2 | Forward | 4956-4983 | TGGAAAGGTGAAGGGGCAGTAGTAATAC |
| B3.R2 | Reverse | 9636-9607 | TGAAGCACTCAAGGCAAGCTTTATTGAGGC |
| **3’ half-genome sequencing primers** | | | |
| Primer name | Primer direction | HXB2 position | Primer sequence (5’🡪3’) |
| BK3.R2 | Reverse | 551-522 | TGAAGCACTCAAGGCAAGCTTTATTGAGGC |
| BK3.F2 | Forward | 4956-4983 | TGGAAAGGTGAAGGGGCAGTAGTAATAC |
| Rev13 | Reverse | 5980-5956 | CTTCCTGCCATAGGAGATGCCTAAG |
| Rev14 | Reverse | 6526-6501 | ACCATGTTATTTTTCCACATGTTAAA |
| Rev15 | Reverse | 7015-6990 | CTGCCATTTAACAGCAGTTGAGTTGA |
| Rev16 | Reverse | 7540-7520 | ATGGGAGGGGCATACATTGCT |
| Rev18 | Reverse | 8365-8341 | GGTGAGTATCCCTGCCTAACTCTAT |
| Rev19 | Reverse | 8820-8797 | ACTTTTTGACCACTTGCCACCCAT |
| For12 | Forward | 5779-5802 | ATTGGGTGCCAACATAGCAGAATA |
| For16 | Forward | 7350-7375 | TTTAATTGTGGAGGAGAATTTTTCTA |
| For17 | Forward | 7796-7819 | AGCAGCAGGAAGCACTATGGGCGC |
| For19 | Forward | 8506-8529 | GGAACCTGTGCCTCTTCAGCTACC |
| B5.R2 | Reverse | 5068-5040 | CAATCATCACCTGCCATCTGTTTTCCATA |
| B3OL.F2 | Forward | 3786-3808 | GAGTGGGAGTTTGTCAATACCCC |
